# Supplementary material for: Learning to Play the Chess Variant Crazyhouse Above World Champion Level With Deep Neural Networks and Human Data
Source: Front Artif Intell. 2020 Apr 28;3:24. doi: 10.3389/frai.2020.00024 (PMC7861260; doi:10.3389/frai.2020.00024)
Supplement: Supplementary file 3 [file Data_Sheet_3.PDF]

Supplementary Material

1 SUPPLEMENTARY DATA

1.1 Matches with 2017 Crazyhouse World Champion Justin Tan (*LM JannLee*)

White: *CrazyAra 0.3.1* Black: *LM JannLee* Result: *1-0* Site: `https://lichess.org/FXeJuDCa`

1. e4 e6 2. Nc3 d5 3. exd5 exd5 4. Bb5+ c6 5. Qe2+ P@e6 6. Bd3 Nd7 7. Nf3 Nc5 8. O-O Nxd3 9. cxd3 Nf6 10. d4 Be7 11. d3 O-O 12. P@h6 gxh6 13. Bxh6 P@g7 14. Bxg7 Kxg7 15. P@g5 Kh8 16. gxf6 Bxf6 17. P@e5 Bg7 18. N@h5 B@h6 19. Nxg7 Bxg7 20. B@f6 Bxf6 21. exf6 Qxf6 22. B@e5 B@g7 23. Bxf6 Bxf6 24. N@h5 B@g7 25. Nxf6 Bxf6 26. B@e5 B@g7 27. Bxf6 Bxf6 28. B@e5 B@g7 29. Nxd5 exd5 30. Bxf6 Bxf6 31. B@e5 B@g7 32. Bxf6 Bxf6 33. B@g5 B@g7 34. P@h6 N@f5 35. hxg7+ Bxg7 36. B@f6 N@h5 37. Bxg7+ Nhxg7 38. B@f6 N@e8 39. Bxg7+ Nexg7 40. Bf6 B@h6 41. N@g4 B@f4 42. Nxh6 Bxh6 43. B@g5 N@h5 44. Bxh6 Nxf6 45. Bxg7+ Nxg7 46. B@h6 Rg8 47. Ng5 B@e6 48. N@e5 B@e8 49. Q@e7 Ngh5 50. Qxh5 Nxh5 51. Qxf7 Rxg5 52. Qxe8+ Q@g8 53. N@f7+ Bxf7 54. Nxf7# 1-0

White: *LM JannLee* Black: *CrazyAra 0.3.1* Result: *1-0* Site: `https://lichess.org/j9eQS4TF`

1. e4 Nc6 2. Nc3 Nf6 3. d4 d5 4. e5 Ne4 5. Bb5 a6 6. Bxc6+ bxc6 7. Nge2 Bf5 8. O-O e6 9. f3 Nxc3 10. bxc3 h5 11. N@e3 N@h4 12. N@a5 B@d7 13. Nxf5 Nxf5 14. B@b7 N@e3 15. Nxc6 Nxd1 16. Nxd8 Rxd8 17. Rxd1 Q@b5 18. Bxa6 Qxa6 19. P@d3 N@e3 20. Bxe3 Nxe3 21. P@d6 Nxd1 22. Rxd1 B@e3+ 23. Kh1 Bxd6 24. exd6 Qxd6 25. B@b4 Qxb4 26. cxb4 P@f2 27. Q@f1 R@g1+ 28. Qxg1 fxg1=Q+ 29. Rxg1 P@f2 30. N@g6 fxg1=Q+ 31. Nxg1 Q@e7 32. Q@d6 fxg6 33. Qxe7+ Kxe7 34. R@f7+ Kxf7 35. N@e5+ Kg8 36. N@f6+ gxf6 37. Q@f7# 1-0

White: *CrazyAra 0.3.1* Black: *LM JannLee* Result: *1-0* Site: `https://lichess.org/CYUSIV9C`

1. e4 e5 2. Nc3 Bc5 3. Nf3 d6 4. Be2 Nf6 5. O-O O-O 6. d3 c6 7. Bg5 Be6 8. d4 exd4 9. Nxd4 Bxd4 10. Qxd4 c5 11. Qxf6 gxf6 12. P@e7 Qxe7 13. N@d5 Q@d8 14. Nxe7+ Qxe7 15. B@g7 Kxg7 16. Q@h6+ Kg8 17. Bxf6 Qxf6 18. Qxf6 P@g7 19. P@h6 B@h8 20. hxg7 Bxg7 21. Qxg7+ Kxg7 22. P@h6+ Kf6 23. B@h4+ P@g5 24. Bxg5+ Kxg5 25. f4+ Kxh6 26. Q@g5# 1-0

White: *LM JannLee* Black: *CrazyAra 0.3.1* Result: *0-1* Site: `https://lichess.org/EeOl3trm`

1. e4 e6 2. d4 d5 3. e5 Nc6 4. Nf3 Nge7 5. Bd3 Nf5 6. Bg5 Be7 7. Bxe7 Qxe7 8. B@c5 Nh4 9. Bxe7 Nxg2+ 10. Kf1 Nxe7 11. Kxg2 P@e4 12. Bb5+ B@d7 13. Be2 exf3+ 14. Bxf3 B@e4 15. Kg1 Bxf3 16. Qxf3 B@e4 17. Qxf7+ Kxf7 18. N@h6+ Ke8 19. P@f7+ Kd8 20. Q@g8+ Q@f8 21. Qxh8 Qxh8 22. R@g8+ N@f8 23. B@f1 gxh6 0-1

White: *CrazyAra 0.3.1* Black: *LM JannLee* Result: *1-0* Site: `https://lichess.org/v1ABi8LI`

1. e4 Nf6 2. Nc3 d5 3. exd5 Nxd5 4. Bb5+ Nc6 5. Nf3 P@h3 6. Rg1 hxg2 7. P@a6 Nxc3 8. bxc3 N@d6 9. axb7 Bxb7 10. P@a6 Nxb5 11. axb7 Rb8 12. B@c4 Nd6 13. Bxf7+ Kxf7 14. Rxg2 B@g6 15. P@h5 Kg8 16. hxg6 hxg6 17. P@e6 B@e8 18. N@g5 Rxb7 19. B@f7+ Nxf7 20. exf7+ Bxf7 21. Nxf7 Qd5 22. Nxh8 Kxh8 23. N@f4 Qe4+ 24. B@e3 N@e5 25. Nxg6+ Qxg6 26. Rxg6 Nxg6 27. P@f7 R@h1+ 28. R@g1 Rxg1+ 29. Nxg1 N@g2+ 30. Kf1 Nxe3+ 31. dxe3 B@c4+ 32. R@d3 Bxf7 33. N@g5 B@g8 34. Nxf7+ Bxf7 35. B@e4 N@e5 36. f4 P@f5 37. Bg2 Ng4 38. Qxg4 fxg4 39. N@g5 Q@g8 40. Q@h5+ R@h6 41. Qxg4 P@b2 42. Bxb2 Rxb2 43. Nxf7+ Qxf7 44. B@e6 B@g8 45. Bxf7 Bxf7 46. P@e6 Bg8 47. P@f7 Bh7 48. Qxg6 Rxg6 49. Q@g8+ Bxg8 50. fxg8=Q+ Kxg8 51. B@f7+ Kh8 52. Bxg6 Q@f2+ 53. Kxf2 Rxc2+ 54. R@d2 B@h4+ 55. N@g3 N@g4+ 56. Kf3 Nxh2+ 57. Ke2 Rxd2+ 58. Kxd2 R@f2+ 59. R@e2 Q@b2+ 60. Q@c2 Qxa1 61. Rxf2 Qxg1 62. R@h7+ Kg8 63. Bf7+ Kxh7 64. Rd8+ 1-0

1.2 Supplementary Figures and Tables

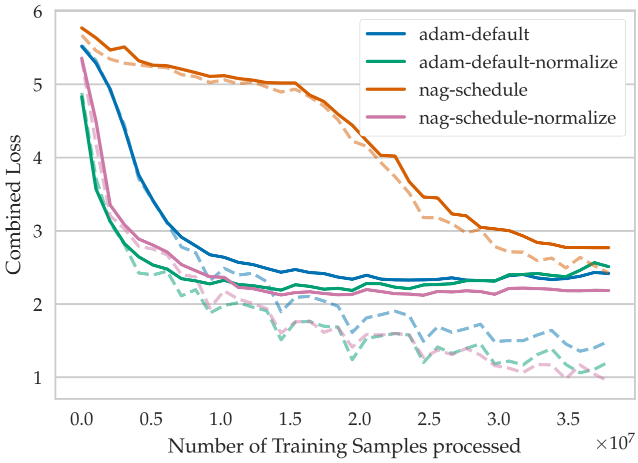

(a) Combined loss

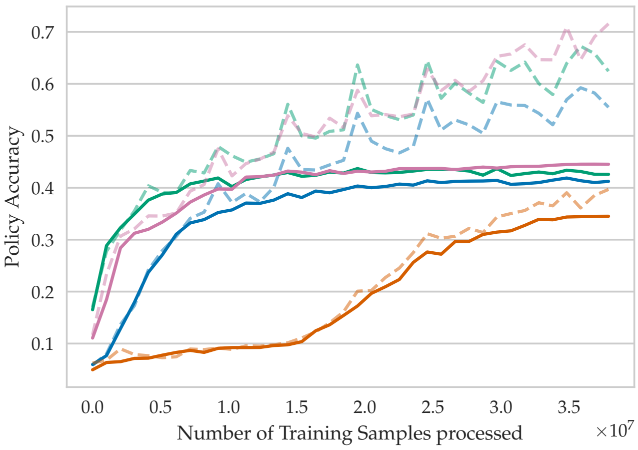

(b) Policy accuracy

Figure S1: Learning progress when training on a subset of the lichess.org data set using 10,000 training games with minimum Elo of 1,600 for both players. The model used was *4-value-8-policy*, 7×256 (Table S2, Table S4). *NAG* stands for the Nesterov Accelerated Gradients. Solid lines and dashed lines describes the score on the validation data set and training set respectively.

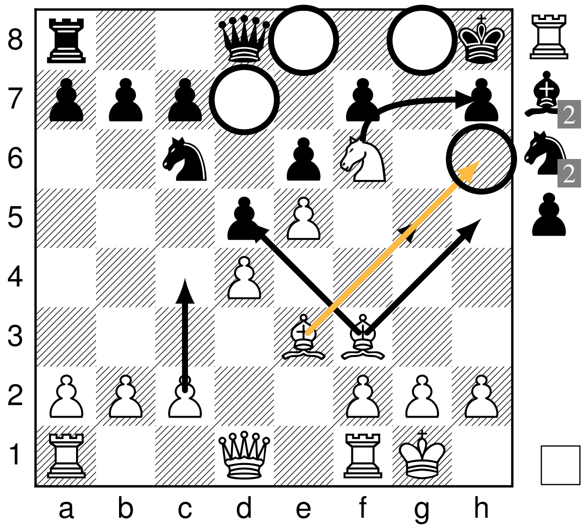

(a) First ten candidate moves

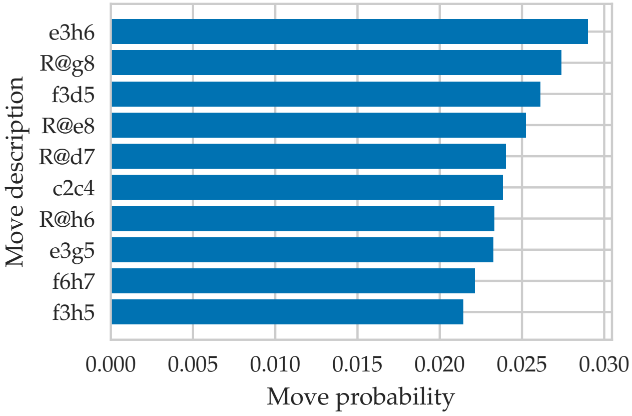

(b) Sorted policy distribution of the first ten candidate moves

Figure S2: Network prediction for the given board position

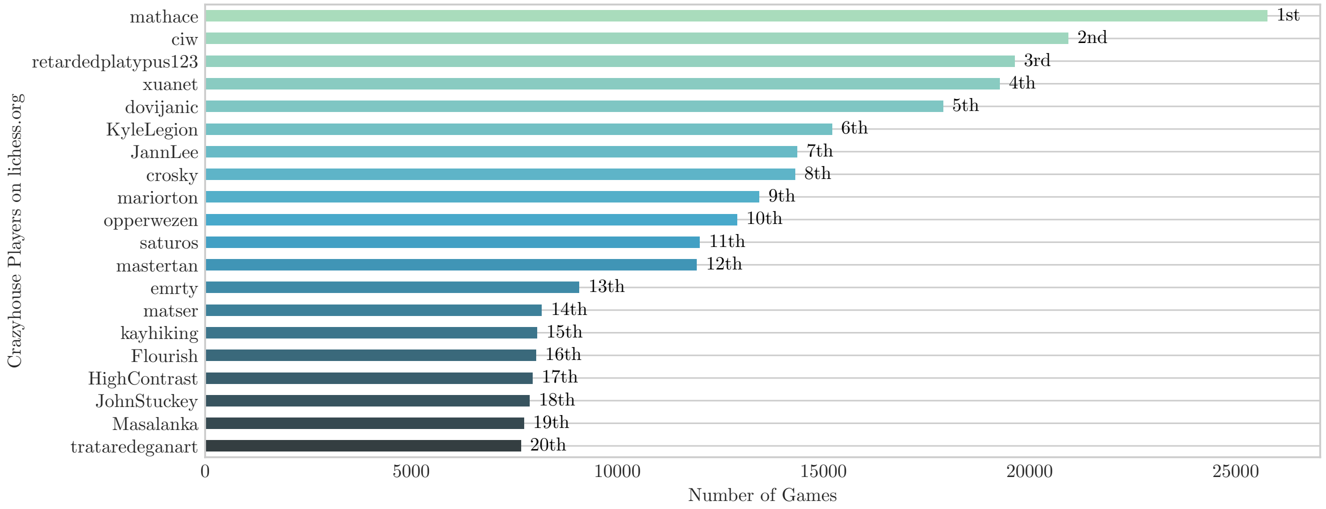

Figure S3: Top 20 most active crazyhouse players with matches $\geq$ 2,000 Elo for both players from January 2016 to June 2018. The training data of *CrazyAra* consists of 569,537 games. These players took part in 46.03 % of all games.

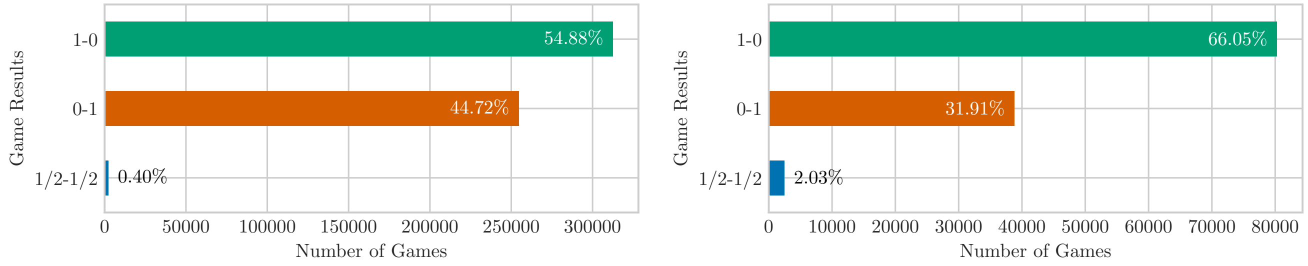

(a) Lichess data set

(b) *Stockfish* self play data set

Figure S4: Game outcomes of the lichess data set (569,537 games) and *Stockfish* self play data set (121,571 games)

Table S1. Policy map representation for crazyhouse

Feature	Planes	Comment
Queen moves	56	direction order: {N, NE, E, SE, S, SW, W, NW} with 7 lengths per direction
Knight moves	8	move order: {2N1E, 1N2E, 1S2E, 2S1E, 2S1W, 1S2W, 1N2W, 2N1W}
Promotions	12	piece order: {KNIGHT, BISHOP, ROOK, QUEEN}
Drop moves	5	piece order: {PAWN, KNIGHT, BISHOP, ROOK, QUEEN}
Total	81	

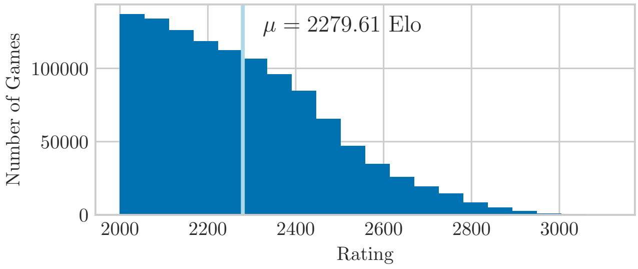

(a) Elo distribution for all selected games

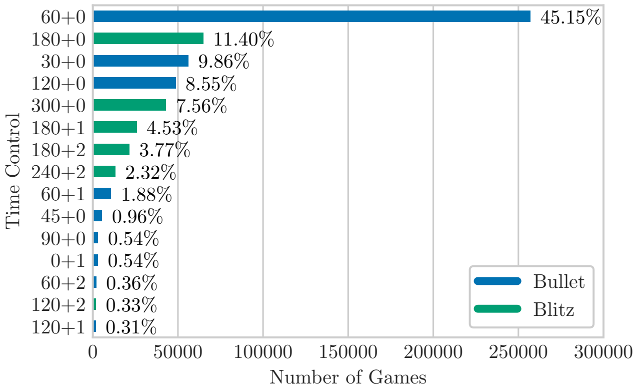

(b) Time control (`total move time[s] + increment[s]`)

Figure S5: Statistics of the lichess data set

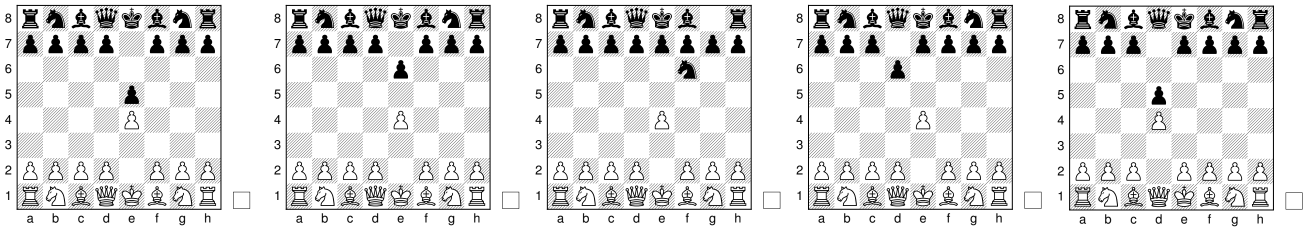

C20 King's Pawn Game | C00 French Defense | B02 Alekhine Defense | B07 Pirc Defense | D00 Queen's Pawn Game

Figure S6: Chosen opening positions validating the playing strength of *CrazyAra 0.6.0* on CPU

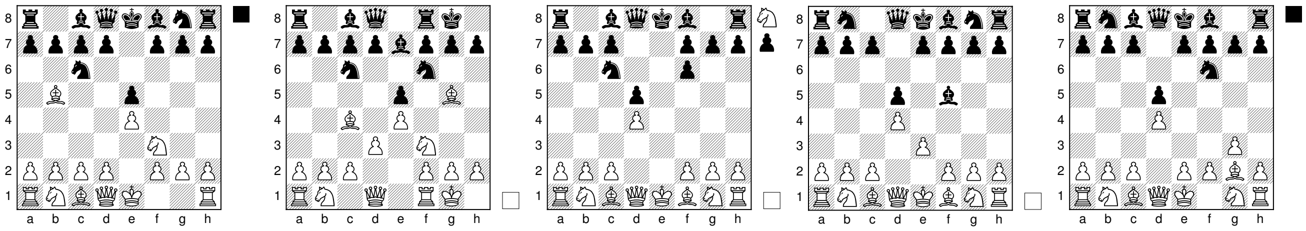

C60 Ruy Lopez | C50 Giuoco Piano | B02 Crosky Gambit | D00 Queen's Pawn Game | A00 Hungarian Opening

Figure S7: Chosen opening positions between *CrazyAraFish 0.6.0* on GPU and *Stockfish 10*

Table S2. *AlphaZero's* network architecture: 19×256

Layer Name	Output Size	AlphaZero Resnet 39-layer
conv0<br>batchnorm0<br>relu0	$256 \times 8 \times 8$	conv $3 \times 3,\ 256$
res_conv0_x<br>res_batchnorm0_x<br>res_relu0_x<br>res_conv1_x<br>res_batchnorm1_x<br>shortcut + output<br>res_relu1_x	$256 \times 8 \times 8$	$\begin{bmatrix} \text{conv } 3 \times 3,\ 256 \\ \text{conv } 3 \times 3,\ 256 \end{bmatrix} \times 19$
value head *policy head*	1 2272 / 5184	Table S4 Table S5 Table S6

Table S3. *8-value-policy-map-preAct-relu+bn*: 19×256

Layer Name	Output Size	AlphaZero Resnet 39-layer (pre-activation)
conv0<br>batchnorm0<br>relu0	$256 \times 8 \times 8$	conv $3 \times 3,\ 256$
res_batchnorm0_x<br>res_conv0_x<br>res_batchnorm1_x<br>res_relu0_x<br>res_conv1_x<br>res_batchnorm2_x<br>shortcut + output	$256 \times 8 \times 8$	$\begin{bmatrix} \text{conv } 3 \times 3,\ 256 \\ \text{conv } 3 \times 3,\ 256 \end{bmatrix} \times 19$
batchnorm1<br>relu1	$256 \times 8 \times 8$	
value head *policy head*	1 2272 / 5184	Table S4 Table S5 Table S6

Table S4. Value head for different architectures with n-channels

Layer Name	Output Size	Value Head N-channels
conv0<br>batchnorm0<br>relu0	$n \times 8 \times 8$	conv $1 \times 1,\ n$
flatten0<br>fully_connected0<br>relu1	256	fc, 256
fully_connected1<br>tanh0	1	fc, 1

Table S5. Policy head type with n-channels

Layer Name	Output Size	Policy Head N-channels
`conv0`<br>`batchnorm0`<br>`relu0`	$n \times 8 \times 8$	conv $1 \times 1,\ n$
`flatten0`<br>`fully_connected0`<br>`softmax0`	2272	fc, 2272

Table S6. Policy head type policy-map

Layer Name	Output Size	Policy Map
`conv0`<br>`batchnorm0`<br>`relu0`	$256 \times 8 \times 8$	conv $3 \times 3,\ 256$
`conv1`<br>`flatten0`<br>`softmax0`	5184	conv $3 \times 3,\ 81$

Table S7. Hyperparameter configuration for supervised learning

Hyperparameter	Value	Comment
Learning rate (max / min)	0.35 / 0.00001	determined by a learning rate range test (Smith and Topin, 2019)
Momentum (min / max)	0.8 / 0.95	recommended by Smith and Topin (2019)
Learning rate schedule / momentum schedule	Linear one cycle schedule	recommended by Smith and Topin (2019)
Epochs	7	determined by training on small subsets of the data set
Batch-Size	$1{,}024$	highest value which fitted in GPU memory (GTX 1080ti)
Optimizer	SGD with Neterov's Momentum (NAG)	comparable to SGD with Momentum used by Silver et al. (2016)
Weight decay	10^{-4}	regularization technique (Silver et al., 2016)
Value loss weighting α	0.01	recommended value for supervised learning by Silver et al. (2016)

Table S8. Hyperparameter configuration of the Monte-Carlo Tree Search

Hyperparameter	Value	Comment
$c_{\text{puct-init}}$	2.5	based on Silver et al. (2017)
$c_{\text{puct-base}}$	19,652	based on Silver et al. (2017)
Batch-Size	8	based on Silver et al. (2017)
Dirichlet ϵ	0.25	based on Silver et al. (2017)
Dirichlet α	0.2	intermediate value for shogi and chess from Silver et al. (2017)
MCTS-Threads	2	each thread thread prepares a single batch
Virtual Loss	3	based on Silver et al. (2017)
u_{init}	1	value of 1 behaves as default
u_{min}	0.25	increases importance for unvisited nodes
u_{base}	1,965	scaling procedure adapted from $c_{\text{puct-base}}$
Q_{factor}	0.7	mixing parameter for node visits and Q-values
$Q_{\text{thresh-init}}$	0.5	Q-value threshold for low node counts
$Q_{\text{thresh-max}}$	0.9	Q-value threshold for high node counts
Transposition table	✓	allows reusing node evaluations
Enhance checking moves	✓	increases probability for checking moves
$check_{\text{thresh}}$	0.1	threshold of low probability checking moves
$check_{\text{factor}}$	0.5	increase factor of checking moves
centi-pawn λ	1.2	conversion parameter for centi-pawn evaluation

Table S9. Average inference time for a batch of size eight for different network architectures and hardware. Time was measured using 300 measurements with 30 preceding warm-up iterations. Lower values are better.

Hardware	Library	4-value-8-policy	8-value-16-policy	8-value-policy-map	8-value-policy-map-mobile	8-value-policy-map-preAct -relu+bn
GeForce GTX 1080 Ti/PCIe/SSE2	MXNet-cu10 1.4.1, CUDA 10.0, cuDNN v7.5.1.10	4.6000ms	4.5986ms	4.8215ms	**3.3500ms**	4.9129ms
AMD® Ryzen 7 1700 eight-core processor × 16	MXNet 1.4.1	263.5504ms	271.8701ms	282.6009ms	**117.5744ms**	279.2975ms
Intel®Core™i5-8250U CPU @ 1.60GHz×8	MXNet 1.4.1	169.0708ms	172.8639ms	176.7654ms	**85.0383ms**	176.1070ms
Intel®Core™i5-8250U CPU @ 1.60GHz×8	MXNet-mkl 1.4.1	93.0978ms	94.7184ms	97.1217ms	**33.7602ms**	96.2892ms
Intel®Core™i5-8250U CPU @ 1.60GHz×8	MXNet-mkl 1.4.1, graph optimization	82.2078ms	83.2606ms	86.5579ms	**28.3759ms**	94.5224ms

Table S10. Match results between Justin Tan and *CrazyAra 0.3.1*

White	Black	Opening	Time Control	Ply Count	Result
CrazyAra 0.3.0	LM JannLee	C00 French Defense	300+15	107	1-0
LM JannLee	CrazyAra 0.3.0	B00 Nimzowitsch Defense	300+15	73	1-0
CrazyAra 0.3.0	LM JannLee	C20 King's Pawn Game	300+15	51	1-0
LM JannLee	CrazyAra 0.3.0	C00 French Defense	300+15	46	0-1
CrazyAra 0.3.0	LM JannLee	B02 Alekhine Defense	300+15	127	1-0

1.3 Matches between *CrazyAraFish 0.6.0* and *Stockfish 10*

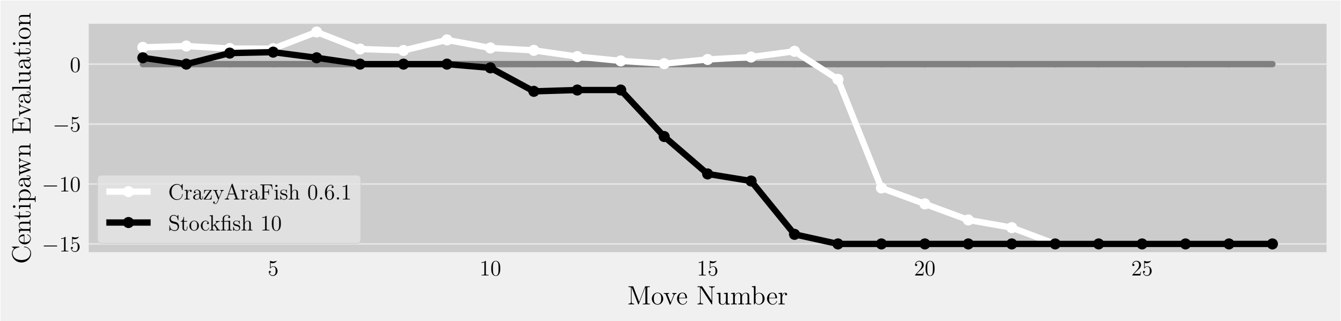

(a) Evaluation progression for both engines

[Event "CrazyAraFish-0.6.0-vs-SF10"]
[Site "Darmstadt, GER"]
[Date "2019.07.26"]
[Round "1"]
[White "CrazyAraFish-0.6.0"]
[Black "stockfish-x86_64-modern 2018-11-29"]
[Result "0-1"]
[PlyCount "58"]
[TimeControl "1800+30"]
[Variant "crazyhouse"]

1. e4 {book} e5 {book} 2. Nf3 {book} Nc6 {book} 3. Bb5 {book} Nf6 {-0.53/29 141s} 4. Nc3 {+1.40/45 59s} Be7 {0.00/27 48s} 5. Nxe5 {+1.51/33 60s} Nxe5 {-0.92/27 83s} 6. d4 {+1.30/43 60s} Ng6 {-1.00/29 187s} 7. e5 {+1.29/56 60s} a6 {-0.53/25 59s} 8. P@h6 {+2.68/52 61s} gxh6 {0.00/23 32s} 9. exf6 {+1.26/40 91s} Bxf6 {0.00/26 100s} 10. Qe2+ {+1.14/48 60s} N@e6 {0.00/28 53s} 11. Bd3 {+2.02/36 60s} d5 {+0.31/24 21s} 12. N@h5 {+1.35/50 90s} Bxd4 {+2.27/22 26s} 13. Bxh6 {+1.15/50 60s} P@e4 {+2.16/24 64s} 14. P@g7 {+0.63/42 60s} Rg8 {+2.16/24 49s} 15. Nxe4 {+0.27/48 60s} P@h3 {+6.04/26 118s} 16. P@f5 {+0.04/53 61s} hxg2 {+9.16/22 26s} 17. Rg1 {+0.39/51 61s} dxe4 {+9.75/24 57s} 18. fxg6 {+0.59/49 61s} N@f3+ {+14.20/23 58s} 19. Qxf3 {+1.07/43 62s} exf3 {+18.23/23 89s} 20. gxh7 {-1.27/51 92s} Q@f1+ {+M21/34 28s} 21. Bxf1 {-10.33/18 91s} P@d2+ {+M19/39 28s} 22. Bxd2 {-11.66/16 60s} Bxf2+ {+M17/42 35s} 23. Kxf2 {-13.00/14 60s} Qxd2+ {+M15/43 50s} 24. B@e2 {-13.64/12 61s} B@d4+ {+M13/44 34s} 25. Kg3 {-16.00/10 61s} P@h4+ {+M9/53 29s} 26. Kxf3 {-18.09/8 61s} Qe3+ {+M7/52 76s} 27. Kg4 {-22.57/6 62s} f5+ {+M5/52 189s} 28. Kxf5 {-29.28/4 62s} Nxg7+ {+M3/61 425s} 29. Kg6 {-M1/2 0.028s} Bf5# {+M1/127 0.009s, Black mates} 0-1

Figure S8: Game 1 / 10

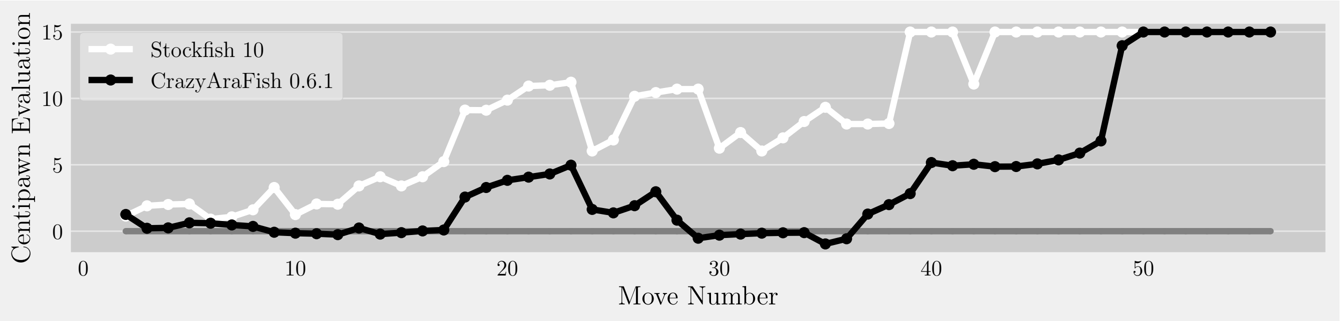

(a) Evaluation progression for both engines

[Event "CrazyAraFish-0.6.0-vs-SF10"]
[Site "Darmstadt, GER"]
[Date "2019.07.26"]
[Round "1"]
[White "stockfish-x86_64-modern 2018-11-29"]
[Black "CrazyAraFish-0.6.0"]
[Result "1-0"]
[PlyCount "115"]
[TimeControl "1800+30"]
[Variant "crazyhouse"]

1. e4 {book} e5 {book} 2. Nf3 {book} Nc6 {book} 3. Bb5 {book} Nf6 {-1.26/33 58s} 4. O-O {+1.14/29 228s} a6 {-0.22/48 59s} 5. Bc4 {+1.91/27 27s} Be7 {-0.25/49 59s} 6. Nc3 {+2.01/29 90s} O-O {-0.63/62 59s} 7. d3 {+2.05/31 120s} d6 {-0.60/60 59s} 8. Ng5 {+0.91/28 80s} Nd4 {-0.47/58 59s} 9. Be3 {+1.08/28 69s} Qe8 {-0.36/56 60s} 10. Ne2 {+1.61/27 38s} Nxe2+ {+0.08/45 60s} 11. Qxe2 {+3.29/23 20s} N@f4 {+0.15/43 61s} 12. Bxf4 {+1.25/31 288s} exf4 {+0.19/42 60s} 13. Rae1 {+2.05/26 26s} B@g4 {+0.26/36 61s} 14. N@f3 {+2.03/27 46s} Kh8 {-0.24/30 61s} 15. N@h4 {+3.41/26 87s} Bce6 {+0.22/29 61s} 16. Bxe6 {+4.10/24 19s} fxe6 {+0.11/36 61s} 17. e5 {+3.41/25 80s} dxe5 {-0.02/50 62s} 18. Qxe5 {+4.10/23 37s} Bxf3 {-0.09/54 62s} 19. P@h6 {+5.24/20 19s} gxh6 {-2.57/48 94s} 20. B@g7+ {+9.12/24 54s} Kxg7 {-3.29/46 61s} 21. Qxe6 {+9.11/24 50s} B@e4 {-3.83/32 62s} 22. Nxe4 {+9.87/26 97s} Qf7 {-4.07/37 62s} 23. Nxf6 {+10.93/25 51s} Bxf6 {-4.31/34 63s} 24. Nxf3 {+10.99/27 117s} Kh8 {-4.97/31 63s} 25. P@e7 {+11.22/26 31s} Qxe6 {-1.64/44 64s} 26. exf8=R+ {+6.04/25 383s} Rxf8 {-1.38/37 63s} 27. Rxe6 {+6.87/25 17s} Q@g8 {-1.92/40 64s} 28. Rxf6 {+10.16/24 57s} Rxf6 {-2.97/52 96s} 29. R@d8 {+10.44/23 15s} B@f8 {-0.83/29 63s} 30. Q@e7 {+10.70/22 18s} N@h3+ {+0.53/50 64s} 31. Kh1 {+10.70/1 0.001s} R@g7 {+0.30/21 64s} 32. Qxf6 {+6.25/25 72s} P@e2 {+0.22/19 64s} 33. Qxg7+ {+7.43/24 43s} Qxg7 {+0.15/17 65s} 34. Rxf8+ {+6.04/24 108s} Qxf8 {+0.12/15 65s} 35. B@d4+ {+7.03/23 26s} P@f6 {+0.11/13 66s} 36. Rg1 {+8.26/25 25s} Q@g7 {+0.96/32 67s} 37. B@h4 {+9.33/24 23s} R@f1 {+0.57/38 67s} 38. R@a8 {+8.07/26 202s} Rxg1+ {-1.29/46 102s} 39. Nxg1 {+8.07/1 0.001s} Qxg2+ {-2.00/32 66s} 40. Kxg2 {+8.11/1 0.001s} R@g7+ {-2.83/36 67s} 41. R@g3 {+18.04/21 27s} Rxg3+ {-5.17/44 67s} 42. fxg3 {+19.81/23 31s} e1=N+ {-4.93/25 42s} 43. Kxh3 {+20.60/21 20s} P@g4+ {-5.04/32 42s} 44. Kxg4 {+11.08/1 0.001s} N@e3+ {-4.86/34 41s} 45. Bxe3 {+20.28/20 20s} R@g7+ {-4.87/36 41s} 46. P@g6 {+21.33/21 66s} h5+ {-5.07/39 40s} 47. Kxf4 {+21.24/22 120s} Qxa8 {-5.37/45 40s} 48. Bc5 {+23.08/19 27s} R@f1+ {-5.88/22 39s} 49. Ke3 {+32.99/19 27s} P@f2 {-6.80/29 39s} 50. N@f7+ {+M17/30 14s} Rxf7 {-13.97/18 57s} 51. g7+ {+M15/35 20s} Rxg7 {-15.49/16 37s} 52. N@g6+ {+M13/45 21s} hxg6 {-17.69/14 37s} 53. Q@h6+ {+M11/50 21s} N@h7 {-19.89/12 36s} 54. Qxg7+ {+M9/63 25s} Kxg7 {-21.18/10 0.048s} 55. R@f7+ {+M7/67 25s} Kxf7 {-23.45/6 37s} 56. R@e7+ {+M5/73 27s} Kg8 {-28.76/4 37s} 57. N@h6+ {+M3/121 20s} Kh8 {-M1/2 18s} 58. P@g7# {+M1/127 0.030s, White mates} 1-0

Figure S9: Game 2 / 10

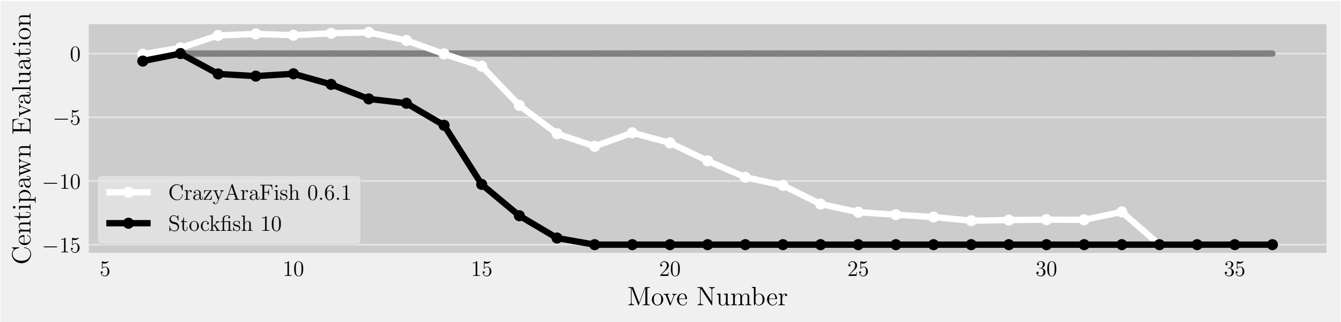

(a) Evaluation progression for both engines

[Event "CrazyAraFish-0.6.0-vs-SF10"]
[Site "Darmstadt, GER"]
[Date "2019.07.26"]
[Round "2"]
[White "CrazyAraFish-0.6.0"]
[Black "stockfish-x86_64-modern 2018-11-29"]
[Result "0-1"]
[PlyCount "74"]
[TimeControl "1800+30"]
[Variant "crazyhouse"]

1. e4 {book} e5 {book} 2. Nf3 {book} Nc6 {book} 3. Bc4 {book} Bc5 {book} 4. O-O {book} Nf6 {book} 5. d3 {book} O-O {book} 6. Bg5 {book} Be7 {book} 7. Nbd2 {-0.05/21 62s} d6 {+0.59/30 128s} 8. Nh4 {+0.45/40 62s} Na5 {0.00/31 153s} 9. Bxf7+ {+1.42/65 63s} Rxf7 {+1.60/24 18s} 10. P@g6 {+1.54/63 62s} hxg6 {+1.77/28 79s} 11. Nxg6 {+1.44/42 63s} B@g4 {+1.59/27 65s} 12. Nxe7+ {+1.59/57 63s} Qxe7 {+2.42/27 43s} 13. B@f3 {+1.66/59 63s} Bxf3 {+3.56/29 171s} 14. Nxf3 {+1.03/48 64s} B@h5 {+3.90/25 16s} 15. B@d5 {-0.03/53 95s} P@e6 {+5.63/26 71s} 16. P@h7+ {-0.99/56 95s} Kxh7 {+10.27/23 17s} 17. d4 {-4.07/32 94s} Bxf3 {+12.73/25 56s} 18. Qxf3 {-6.29/32 93s} P@g4 {+14.48/22 24s} 19. Qe3 {-7.28/28 61s} exd4 {+17.87/24 60s} 20. Bxf6 {-6.22/34 62s} gxf6 {+18.66/23 39s} 21. B@f5+ {-7.01/35 62s} exf5 {+24.99/23 25s} 22. exf5 {-8.42/28 62s} dxe3 {+27.95/24 36s} 23. P@g6+ {-9.71/28 62s} Kg7 {+29.84/24 52s} 24. gxf7 {-10.35/32 62s} N@h3+ {+30.87/23 35s} 25. gxh3 {-11.81/34 32s} N@e2+ {+32.14/22 48s} 26. Kh1 {-12.45/33 64s} B@e4+ {+32.05/23 76s} 27. N@g2 {-12.65/32 65s} Bxd5 {+35.01/24 66s} 28. R@g8+ {-12.83/30 65s} Kh7 {+35.36/24 35s} 29. Rxg4 {-13.12/29 66s} Bxf5 {+36.37/22 41s} 30. P@g6+ {-13.07/29 66s} Bxg6 {+37.67/23 111s} 31. Rxg6 {-13.04/29 67s} Kxg6 {+38.08/22 30s} 32. N@h4+ {-13.05/27 67s} Kg7 {+41.31/21 53s} 33. B@h6+ {-12.43/14 68s} Kxh6 {+M21/21 36s} 34. f3 {-15.63/19 68s} Qe5 {+M13/40 34s} 35. Nxe3 {-20.14/6 69s} Qxh2+ {+M5/68 92s} 36. Kxh2 {-24.74/4 0.12s} P@g3+ {+M3/109 36s} 37. Kh1 {-M1/2 75s} R@h2# {+M1/127 0.024s, Black mates} 0-1

Figure S10: Game 3 / 10

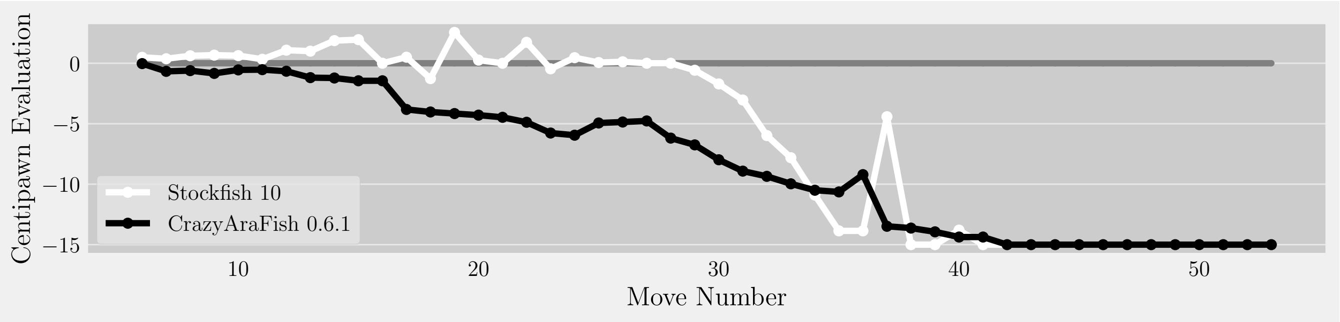

(a) Evaluation progression for both engines

[Event "CrazyAraFish-0.6.0-vs-SF10"]
[Site "Darmstadt, GER"]
[Date "2019.07.26"]
[Round "2"]
[White "stockfish-x86_64-modern 2018-11-29"]
[Black "CrazyAraFish-0.6.0"]
[Result "0-1"]
[PlyCount "108"]
[TimeControl "1800+30"]
[Variant "crazyhouse"]

1. e4 {book} e5 {book} 2. Nf3 {book} Nc6 {book} 3. Bc4 {book} Bc5 {book} 4. O-O {book} Nf6 {book} 5. d3 {book} O-O {book} 6. Bg5 {book} Be7 {book} 7. Be3 {+0.49/28 118s} d6 {+0.03/35 62s} 8. a3 {+0.37/31 164s} Kh8 {+0.67/38 62s} 9. Kh1 {+0.62/27 23s} Bg4 {+0.61/37 62s} 10. Nbd2 {+0.67/29 54s} Nd4 {+0.84/49 63s} 11. Rg1 {+0.63/30 230s} c6 {+0.55/31 63s} 12. c3 {+0.33/31 88s} Ne6 {+0.53/35 63s} 13. h3 {+1.08/25 16s} Bxf3 {+0.66/38 63s} 14. Nxf3 {+1.00/28 102s} N@g4 {+1.19/39 64s} 15. hxg4 {+1.87/25 27s} Nxg4 {+1.22/33 64s} 16. B@f5 {+1.95/30 349s} Nxe3 {+1.45/31 64s} 17. fxe3 {0.00/30 100s} d5 {+1.45/53 64s} 18. Bb3 {+0.50/25 123s} Ng5 {+3.82/33 65s} 19. exd5 {-1.27/24 51s} Nxf3 {+4.03/39 65s} 20. Qxf3 {+2.55/25 25s} N@h4 {+4.16/37 65s} 21. Qg4 {+0.26/29 343s} Nxf5 {+4.29/35 66s} 22. Qxf5 {0.00/25 28s} cxd5 {+4.47/34 66s} 23. P@h6 {+1.73/25 72s} gxh6 {+4.88/38 66s} 24. Qxe5+ {-0.46/27 236s} B@f6 {+5.77/33 67s} 25. Qh5 {+0.46/25 23s} P@g7 {+5.95/37 67s} 26. P@f2 {+0.06/27 78s} B@e6 {+4.94/35 67s} 27. N@f4 {+0.12/29 47s} Bd6 {+4.86/38 67s} 28. Qe2 {0.00/28 39s} Bxf4 {+4.77/30 68s} 29. exf4 {0.00/25 13s} P@h3 {+6.19/34 68s} 30. gxh3 {-0.58/25 50s} Bxh3 {+6.75/31 69s} 31. P@g2 {-1.71/28 97s} Bxg2+ {+7.98/33 69s} 32. Rxg2 {-3.04/21 23s} P@h3 {+8.92/36 69s} 33. Rg3 {-5.98/23 58s} Re8 {+9.35/36 70s} 34. B@e6 {-7.81/22 32s} Rxe6 {+9.97/34 71s} 35. Qxe6 {-10.90/21 30s} B@g2+ {+10.50/27 71s} 36. Kh2 {-13.86/21 30s} N@f3+ {+10.64/31 72s} 37. Rxf3 {-13.86/1 0s} Bxf3 {+9.21/43 72s} 38. N@e3 {-4.42/22 27s} R@g2+ {+13.48/28 73s} 39. Nxg2 {-20.48/24 60s} hxg2 {+13.63/23 74s} 40. B@e2 {-21.62/23 33s} Bxe2 {+13.94/23 75s} 41. Qxe2 {-13.80/19 7.6s} B@f1 {+14.37/22 48s} 42. Qf3 {-18.02/21 39s} Qd7 {+14.37/21 48s} 43. f5 {-32.92/18 43s} N@e5 {+17.28/13 47s} 44. Qg3 {-30.51/17 30s} P@h4 {+20.17/9 46s} 45. Qxg2 {-M16/28 30s} Bxg2 {+22.24/18 45s} 46. Kxg2 {-M14/30 9.6s} Qxf5 {+28.03/11 44s} 47. R@b8+ {-M12/30 15s} Rxb8 {+35.36/11 22s} 48. R@g8+ {-M10/29 16s} Rxg8 {+35.81/17 44s} 49. N@g6+ {-M8/46 21s} hxg6 {+34.93/15 43s} 50. N@g1 {-M6/51 16s} P@f3+ {+37.14/7 42s} 51. Kf1 {-M6/85 17s} P@g2+ {+37.45/5 42s} 52. Ke1 {-M4/1 0.001s} Qxd3 {+55.96/3 41s} 53. N@d2 {-M4/67 13s} R@f1+ {+85.21/3 41s} 54. Nxf1 {-M2/1 0.001s} gxf1=Q# {+M1/1 60s, Black mates} 0-1

Figure S11: Game 4 / 10

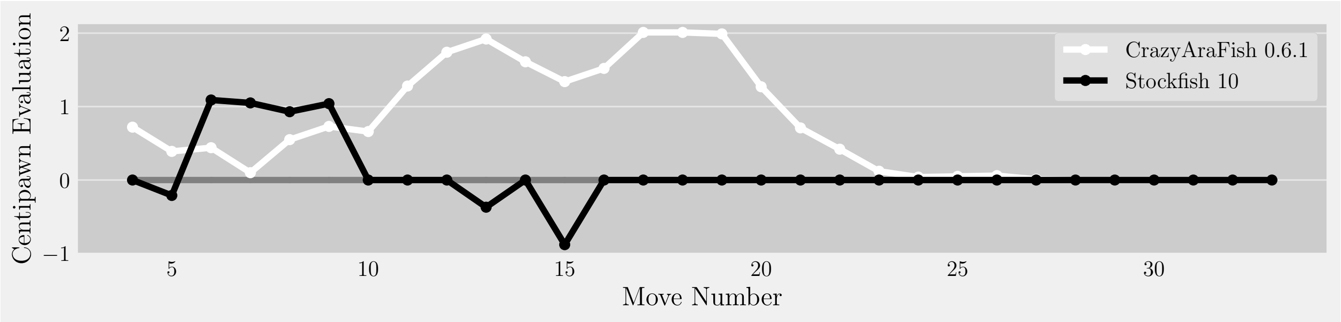

(a) Evaluation progression for both engines

[Event "CrazyAraFish-0.6.0-vs-SF10"]
[Site "Darmstadt, GER"]
[Date "2019.07.26"]
[Round "3"]
[White "CrazyAraFish-0.6.0"]
[Black "stockfish-x86_64-modern 2018-11-29"]
[Result "1/2-1/2"]
[PlyCount "68"]
[TimeControl "1800+30"]
[Variant "crazyhouse"]

1. e4 {book} Nf6 {book} 2. e5 {book} Nc6 {book} 3. exf6 {book} exf6 {book} 4. d4 {book} d5 {book} 5. a3 {+0.72/38 60s} Bd6 {0.00/29 127s} 6. Nc3 {+0.39/49 60s} Be6 {+0.21/26 86s} 7. Nf3 {+0.44/40 61s} O-O {-1.09/27 102s} 8. N@h5 {+0.10/44 61s} Re8 {-1.05/26 103s} 9. Be2 {+0.55/41 61s} Bf8 {-0.93/25 33s} 10. Be3 {+0.73/42 61s} Ne7 {-1.04/27 67s} 11. O-O {+0.66/22 62s} Nf5 {0.00/27 34s} 12. Bf4 {+1.28/21 62s} P@e4 {0.00/28 29s} 13. Ne1 {+1.74/41 62s} c6 {0.00/29 57s} 14. Kh1 {+1.92/46 62s} Qb6 {+0.37/29 92s} 15. Bg4 {+1.61/57 62s} Nxd4 {0.00/30 252s} 16. Be3 {+1.34/55 63s} Bxg4 {+0.88/24 17s} 17. Qxg4 {+1.52/43 63s} P@g6 {0.00/30 73s} 18. Nxg7 {+2.01/45 64s} Bxg7 {0.00/32 38s} 19. P@d7 {+2.01/37 63s} Red8 {0.00/32 82s} 20. Nxe4 {+1.99/47 64s} dxe4 {0.00/26 26s} 21. P@e7 {+1.27/43 64s} B@f5 {0.00/31 35s} 22. exd8=R+ {+0.71/41 65s} Qxd8 {0.00/32 34s} 23. R@e8+ {+0.42/39 65s} Qxe8 {0.00/33 35s} 24. dxe8=R+ {+0.12/62 65s} Rxe8 {0.00/33 59s} 25. Bxd4 {+0.04/16 66s} Bxg4 {0.00/33 79s} 26. Bxf6 {+0.05/14 66s} Q@h5 {0.00/32 69s} 27. Bxg7 {+0.06/12 66s} N@g3+ {0.00/34 80s} 28. fxg3 {+0.01/10 66s} R@g1+ {0.00/35 100s} 29. Kxg1 {0.00/8 67s} N@e2+ {0.00/33 138s} 30. Kh1 {0.00/6 67s} Nxg3+ {0.00/35 375s} 31. Kg1 {0.00/4 0.005s} Ne2+ {0.00/36 25s} 32. Kh1 {0.00/2 72s} Ng3+ {0.00/38 221s} 33. Kg1 {0.00/1 0.006s} Ne2+ {0.00/36 125s} 34. Kh1 {0.00/1 77s} Ng3+ {0.00/40 22s, Draw by 3-fold repetition} 1/2-1/2

Figure S12: Game 5 / 10

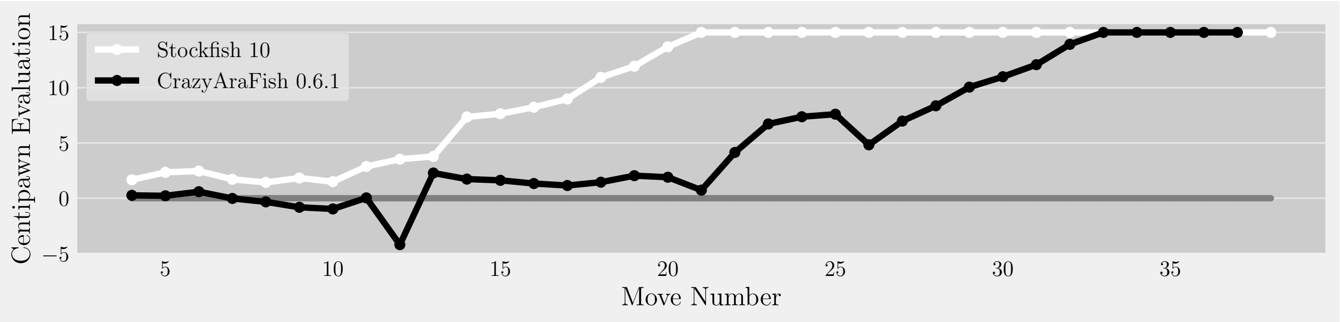

(a) Game 6 / 10: Evaluation progression for both engines

[Event "CrazyAraFish-0.6.0-vs-SF10"]
[Site "Darmstadt, GER"]
[Date "2019.07.26"]
[Round "3"]
[White "stockfish-x86_64-modern 2018-11-29"]
[Black "CrazyAraFish-0.6.0"]
[Result "1-0"]
[PlyCount "77"]
[TimeControl "1800+30"]
[Variant "crazyhouse"]

1. e4 {book} Nf6 {book} 2. e5 {book} Nc6 {book} 3. exf6 {book} exf6 {book} 4. d4 {book} d5 {book} 5. a3 {+1.67/28 126s} Be7 {-0.26/38 60s} 6. Nc3 {+2.34/26 78s} O-O {-0.22/51 60s} 7. Be2 {+2.46/27 37s} Re8 {-0.59/50 61s} 8. Nf3 {+1.71/28 124s} Bf8 {+0.02/45 61s} 9. Be3 {+1.43/30 388s} Ne7 {+0.33/34 61s} 10. N@h4 {+1.83/26 20s} P@e4 {+0.82/28 61s} 11. Nd2 {+1.50/28 66s} g5 {+0.97/40 62s} 12. Ndxe4 {+2.87/28 45s} dxe4 {-0.03/46 92s} 13. Bc4 {+3.53/26 43s} N@d6 {+4.20/40 61s} 14. Nxe4 {+3.78/25 34s} Nef5 {-2.28/36 92s} 15. Nxf5 {+7.35/24 26s} Bxf5 {-1.73/43 61s} 16. Nxd6 {+7.64/25 24s} cxd6 {-1.62/29 61s} 17. N@g3 {+8.23/25 76s} N@h4 {-1.33/38 62s} 18. O-O {+8.98/25 28s} N@h3+ {-1.15/43 61s} 19. gxh3 {+10.93/25 19s} Bxh3 {-1.45/47 62s} 20. Bxf7+ {+11.94/24 44s} Kxf7 {-2.04/43 62s} 21. d5 {+13.70/24 61s} P@g2 {-1.90/37 63s} 22. Re1 {+15.03/25 30s} Qa5 {-0.73/42 63s} 23. b4 {+18.24/21 32s} Qb5 {-4.14/40 94s} 24. P@e6+ {+20.23/23 50s} Rxe6 {-6.72/38 93s} 25. dxe6+ {+21.29/19 33s} Bxe6 {-7.37/36 61s} 26. P@e7 {+25.00/19 38s} P@d2 {-7.60/34 62s} 27. Bxd2 {+31.57/21 70s} P@e2 {-4.83/26 63s} 28. exf8=R+ {+41.73/22 99s} Kxf8 {-6.98/32 94s} 29. P@e7+ {+44.38/20 25s} Kxe7 {-8.36/26 61s} 30. Qxe2 {+46.30/20 48s} Qxe2 {-10.05/30 62s} 31. N@d5+ {+M19/27 27s} Kf8 {-10.99/28 62s} 32. P@e7+ {+M17/31 31s} Kf7 {-12.08/26 63s} 33. R@f8+ {+M15/35 44s} Rxf8 {-13.92/18 63s} 34. exf8=Q+ {+M13/44 32s} Kxf8 {-15.47/16 32s} 35. P@e7+ {+M11/51 42s} Kg7 {-17.56/8 66s} 36. e8=N+ {+M7/61 158s} Kf7 {-22.48/6 67s} 37. R@e7+ {+M5/65 35s} Kg6 {-31.00/4 67s} 38. N@f8+ {+M3/71 74s} Kh6 {-M1/2 0.040s} 39. Rxh7# {+M1/127 0.010s, White mates} 1-0

Figure S13: Game 6 / 10

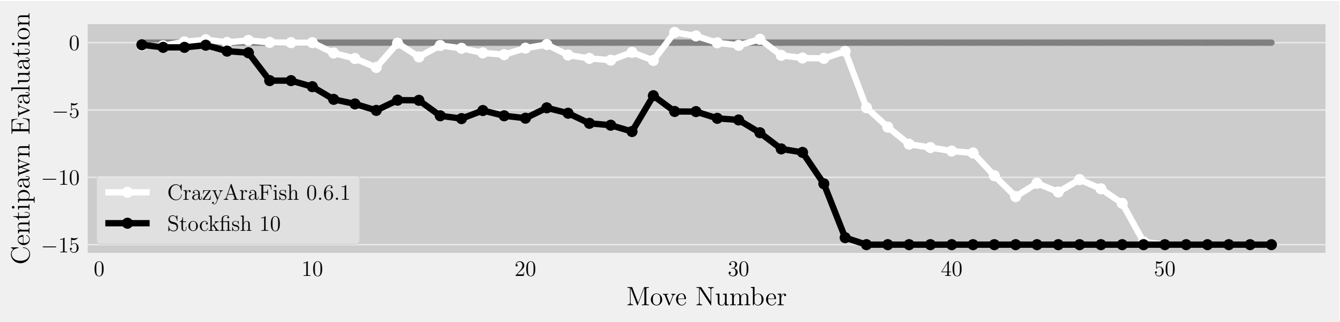

(a) Evaluation progression for both engines

[Event "CrazyAraFish-0.6.0-vs-SF10"]
[Site "Darmstadt, GER"]
[Date "2019.07.26"]
[Round "4"]
[White "CrazyAraFish-0.6.0"]
[Black "stockfish-x86_64-modern 2018-11-29"]
[Result "0-1"]
[PlyCount "112"]
[TimeControl "1800+30"]
[Variant "crazyhouse"]

1. e3 {book} d5 {book} 2. d4 {book} Bf5 {book} 3. Bd3 {-0.22/23 58s} e6 {+0.16/31 175s} 4. Nc3 {-0.23/23 59s} Bxd3 {+0.35/31 130s} 5. cxd3 {+0.06/31 30s} Nf6 {+0.35/26 17s} 6. Nf3 {+0.22/25 60s} B@g4 {+0.19/27 125s} 7. B@e2 {+0.02/31 60s} Bd6 {+0.63/25 15s} 8. Ne5 {+0.17/42 60s} Bxe2 {+0.75/27 54s} 9. Nxe2 {+0.02/37 60s} Nc6 {+2.82/27 47s} 10. B@g3 {0.00/34 61s} Bxe5 {+2.82/26 32s} 11. dxe5 {0.00/28 61s} Ng4 {+3.27/26 78s} 12. Nd4 {-0.77/34 92s} Ngxe5 {+4.21/24 29s} 13. Nxc6 {-1.18/34 61s} Nxc6 {+4.55/27 96s} 14. O-O {-1.84/28 61s} O-O {+5.03/25 54s} 15. N@h5 {-0.04/37 61s} B@e4 {+4.27/26 287s} 16. f3 {-1.06/40 92s} Bxf3 {+4.28/27 152s} 17. Qxf3 {-0.21/32 61s} N@g5 {+5.43/27 23s} 18. Qe2 {-0.42/41 61s} P@e4 {+5.64/24 16s} 19. B@f6 {-0.76/44 61s} P@f3 {+5.04/26 74s} 20. gxf3 {-0.89/44 61s} exf3 {+5.43/30 132s} 21. Rxf3 {-0.41/52 62s} N@h3+ {+5.61/29 148s} 22. Kh1 {-0.14/37 62s} Nxf3 {+4.84/28 76s} 23. Bd2 {-0.92/38 94s} Nxd2 {+5.24/26 54s} 24. B@g4 {-1.17/37 61s} gxf6 {+5.99/25 31s} 25. P@g7 {-1.30/35 62s} B@h6 {+6.13/25 18s} 26. gxf8=Q+ {-0.72/37 63s} Bxf8 {+6.60/26 79s} 27. R@e8 {-1.32/19 63s} Qxe8 {+3.94/26 163s} 28. Nxf6+ {+0.76/20 63s} Kh8 {+5.11/24 29s} 29. Nxe8 {+0.50/40 63s} R@g6 {+5.12/25 30s} 30. Q@c3+ {-0.01/43 96s} B@e5 {+5.62/26 64s} 31. Qcxd2 {-0.20/28 63s} Rxe8 {+5.74/25 75s} 32. N@h4 {+0.25/44 63s} N@f2+ {+6.69/23 21s} 33. Bxf2 {-0.94/37 95s} Nxf2+ {+7.89/25 47s} 34. Qxf2 {-1.14/35 31s} Rxg4 {+8.15/24 26s} 35. P@g7+ {-1.17/40 65s} Bexg7 {+10.48/25 108s} 36. P@g6 {-0.65/44 66s} hxg6 {+14.48/22 25s} 37. Qxf7 {-4.83/30 99s} Rxh4 {+21.37/22 18s} 38. Qff2 {-6.27/22 96s} B@f3+ {+25.59/24 37s} 39. Qxf3 {-7.54/28 62s} Ne5 {+25.80/22 13s} 40. B@f6 {-7.79/27 63s} Nxf3 {+36.99/25 28s} 41. N@f7+ {-8.05/30 43s} Kg8 {+39.41/25 47s} 42. Bxh4 {-8.19/33 42s} P@h3 {+41.51/23 62s} 43. Nh6+ {-9.88/25 41s} Kh7 {+44.90/19 23s} 44. P@g2 {-11.43/20 41s} Q@h5 {+49.26/20 65s} 45. R@f2 {-10.44/24 40s} hxg2+ {+55.72/19 25s} 46. Rxg2 {-11.09/22 40s} Nxd2 {+M35/21 51s} 47. N@g5+ {-10.17/18 39s} Kxh6 {+M29/21 18s} 48. Rxd2 {-10.85/28 39s} Qxh4 {+M21/25 39s} 49. P@g3 {-11.93/14 38s} P@h3 {+M17/31 32s} 50. N@g8+ {-14.79/14 38s} Kh5 {+M15/39 18s} 51. gxh4 {-16.80/10 37s} B@f3+ {+M11/42 19s} 52. Nxf3 {-17.47/10 37s} P@g2+ {+M9/51 31s} 53. Rxg2 {-20.33/8 37s} hxg2+ {+M7/55 100s} 54. Kxg2 {-22.83/6 36s} P@h3+ {+M5/57 75s} 55. Kf1 {-26.40/4 36s} Q@g2+ {+M3/59 32s} 56. Ke1 {-M1/2 0.028s} R@f1# {+M1/127 0.022s, Black mates} 0-1

Figure S14: Game 7 / 10

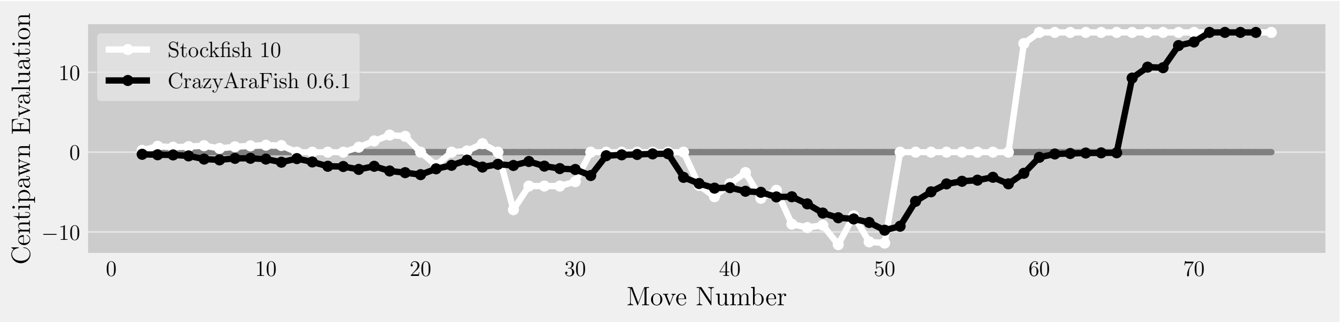

(a) Evaluation progression for both engines

[Event "CrazyAraFish-0.6.0-vs-SF10"]
[Site "Darmstadt, GER"]
[Date "2019.07.27"]
[Round "4"]
[White "stockfish-x86_64-modern 2018-11-29"]
[Black "CrazyAraFish-0.6.0"]
[Result "1-0"]
[PlyCount "151"]
[TimeControl "1800+30"]
[Variant "crazyhouse"]

1. e3 {book} d5 {book} 2. d4 {book} Bf5 {book} 3. Bd3 {+0.22/31 173s} e6 {+0.27/24 58s} 4. Bxf5 {+0.73/27 31s} exf5 {+0.33/23 59s} 5. Ne2 {+0.62/30 276s} Nf6 {+0.35/25 59s} 6. Nbc3 {+0.69/26 25s} Nc6 {+0.47/23 59s} 7. B@h4 {+0.81/29 190s} Be7 {+0.87/29 60s} 8. O-O {+0.47/28 169s} O-O {+0.97/41 60s} 9. Bd2 {+0.67/28 40s} B@d6 {+0.78/32 60s} 10. Ng3 {+0.79/25 19s} Ne4 {+0.77/28 60s} 11. Bxe7 {+0.87/27 45s} Nxe7 {+0.87/32 60s} 12. B@e5 {+0.83/29 157s} B@h6 {+1.26/26 61s} 13. Ncxe4 {0.00/29 112s} dxe4 {+0.81/45 61s} 14. Qh5 {0.00/29 52s} Bxe5 {+1.23/47 61s} 15. dxe5 {0.00/29 44s} B@f3 {+1.77/49 61s} 16. gxf3 {0.00/29 24s} exf3 {+1.80/47 61s} 17. Bc3 {+0.62/24 203s} Nd5 {+2.16/40 62s} 18. B@h3 {+1.40/23 28s} Nxc3 {+1.77/40 62s} 19. bxc3 {+2.14/23 14s} N@e2+ {+2.36/38 62s} 20. Nxe2 {+1.98/25 59s} fxe2 {+2.57/38 62s} 21. Qxe2 {0.00/29 65s} P@g4 {+2.82/35 63s} 22. B@f4 {-1.65/28 323s} Bxf4 {+2.09/34 64s} 23. exf4 {0.00/22 15s} N@f3+ {+1.65/42 63s} 24. Qxf3 {+0.15/23 19s} gxf3 {+1.00/34 64s} 25. B@h1 {+1.06/26 53s} B@g4 {+1.87/45 64s} 26. Bxg4 {0.00/26 156s} fxg4 {+1.52/44 64s} 27. B@f6 {-7.21/22 117s} B@g3 {+1.67/65 65s} 28. hxg3 {-4.25/25 21s} B@h2+ {+1.17/63 65s} 29. Kxh2 {-4.25/1 0.001s} Q@h3+ {+1.75/61 65s} 30. Kg1 {-4.25/1 0s} Qxf6 {+2.06/55 66s} 31. N@h2 {-3.70/25 14s} Qxf4 {+2.17/52 66s} 32. N@e7+ {0.00/26 43s} Kh8 {+2.93/21 1.0s} 33. P@f6 {0.00/26 37s} B@h6 {+0.45/44 106s} 34. fxg7+ {0.00/27 49s} Bxg7 {+0.34/42 35s} 35. P@f6 {0.00/29 18s} Qxe5 {+0.29/40 72s} 36. B@d4 {0.00/29 23s} Qxf6 {+0.22/38 73s} 37. Bxf6 {0.00/30 97s} Bxf6 {+0.20/36 73s} 38. B@h4 {0.00/30 45s} Bxe7 {+3.16/42 75s} 39. Bxf3 {-4.17/23 34s} B@g7 {+3.93/32 75s} 40. P@f6 {-5.58/26 98s} Bexf6 {+4.51/40 76s} 41. Bxf6 {-3.96/24 22s} Bxf6 {+4.45/38 49s} 42. Q@f4 {-2.56/23 47s} B@e5 {+4.90/36 48s} 43. Qxg4 {-5.78/22 29s} Qxg4 {+5.03/31 47s} 44. Nxg4 {-4.78/21 26s} P@h2+ {+5.61/28 46s} 45. Nxh2 {-9.02/19 49s} P@e2 {+5.59/32 46s} 46. Q@g2 {-9.45/20 30s} P@h3 {+6.48/26 45s} 47. Qxh3 {-9.15/19 8.5s} N@g5 {+7.63/27 44s} 48. Qg4 {-11.59/22 52s} Nxf3+ {+8.22/27 43s} 49. Qxf3 {-7.98/20 7.0s} exf1=R+ {+8.37/30 42s} 50. Rxf1 {-11.24/25 53s} P@h3 {+8.81/23 42s} 51. B@h1 {-11.39/23 30s} B@c6 {+9.78/25 41s} 52. Qxf6+ {0.00/25 12s} Bxf6 {+9.28/22 41s} 53. B@g5 {0.00/28 32s} P@g7 {+6.16/39 60s} 54. Bxf6 {0.00/28 19s} Q@g6 {+4.97/33 39s} 55. Bxg7+ {0.00/29 20s} Qxg7 {+3.98/31 38s} 56. P@f6 {0.00/30 36s} Qxf6 {+3.65/36 38s} 57. B@e7 {0.00/29 43s} Q@g5 {+3.51/39 37s} 58. Bxf6+ {0.00/30 25s} Qxf6 {+3.15/37 37s} 59. Q@f5 {0.00/30 24s} B@e5 {+3.97/25 37s} 60. P@h6 {+13.61/27 58s} P@g7 {+2.65/38 54s} 61. hxg7+ {+16.60/22 15s} Qxg7 {+0.65/19 18s} 62. P@h6 {+19.05/24 41s} Qxh6 {+0.23/17 36s} 63. Qxe5+ {+20.50/21 10s} P@g7 {+0.17/15 35s} 64. P@f6 {+28.84/22 23s} gxf6 {+0.11/13 35s} 65. B@g5 {+37.64/21 17s} B@g7 {+0.09/11 35s} 66. Bxh6 {+47.40/23 13s} Bxh6 {+0.08/9 35s} 67. N@g5 {+M25/20 20s} R@g6 {-9.28/28 52s} 68. P@g7+ {+M19/28 14s} Rxg7 {-10.65/20 33s} 69. Qxf6 {+M15/31 17s} B@g6 {-10.58/18 33s} 70. N@e7 {+M13/38 20s} Bxg5 {-13.35/12 33s} 71. Qxg7+ {+M11/48 22s} Kxg7 {-13.80/10 0.037s} 72. N@h5+ {+M9/50 58s} Bxh5 {-19.59/8 34s} 73. Nf5+ {+M7/54 30s} Kf6 {-23.11/6 34s} 74. Q@e7+ {+M5/59 18s} Kg6 {-30.89/4 34s} 75. R@g7+ {+M3/65 25s} Kxf5 {-M1/2 0.022s} 76. Rxg5# {+M1/127 0.017s, White mates} 1-0

Figure S15: Game 8 / 10

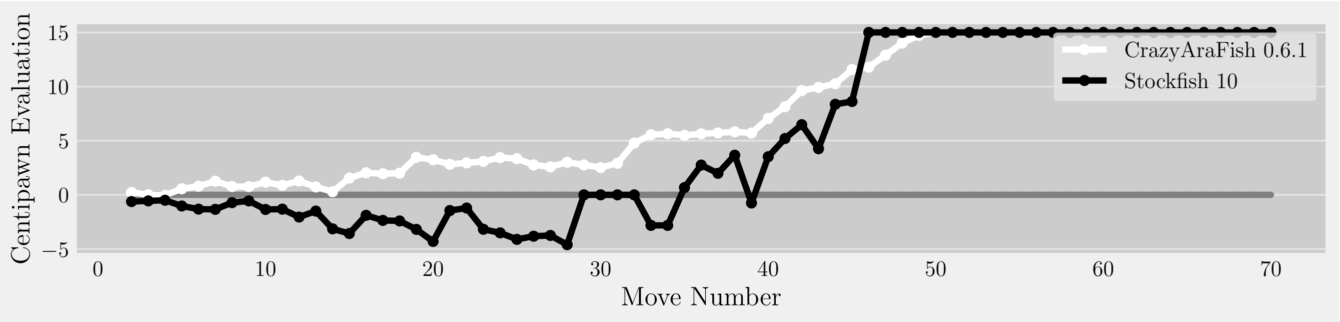

(a) Evaluation progression for both engines

[Event "CrazyAraFish-0.6.0-vs-SF10"]
[Site "Darmstadt, GER"]
[Date "2019.07.27"]
[Round "5"]
[White "CrazyAraFish-0.6.0"]
[Black "stockfish-x86_64-modern 2018-11-29"]
[Result "1-0"]
[PlyCount "143"]
[TimeControl "1800+30"]
[Variant "crazyhouse"]

1. g3 {book} d5 {book} 2. d4 {book} Nf6 {book} 3. Bg2 {book} Bf5 {+0.62/30 139s} 4. Nf3 {+0.22/38 59s} e6 {+0.56/28 47s} 5. Nc3 {+0.01/37 60s} Be7 {+0.50/29 33s} 6. Nh4 {-0.04/29 60s} Be4 {+1.03/28 58s} 7. Nxe4 {+0.56/23 60s} Nxe4 {+1.32/25 18s} 8. O-O {+0.80/21 60s} O-O {+1.34/26 57s} 9. B@e5 {+1.26/32 61s} f6 {+0.72/29 254s} 10. Bef4 {+0.77/47 61s} f5 {+0.56/27 43s} 11. f3 {+0.76/35 61s} Nd6 {+1.35/29 58s} 12. Bxd6 {+1.17/27 61s} Qxd6 {+1.32/28 32s} 13. N@f4 {+0.88/43 61s} Nc6 {+2.05/27 63s} 14. Kh1 {+1.28/32 62s} B@e8 {+1.50/29 239s} 15. Be3 {+0.73/41 62s} N@c4 {+3.14/24 24s} 16. Qc1 {+0.29/42 62s} Nxe3 {+3.57/27 63s} 17. Qxe3 {+1.55/48 62s} Nxd4 {+1.89/28 89s} 18. Qxd4 {+2.04/34 63s} B@b6 {+2.36/28 73s} 19. Qxg7+ {+1.95/41 63s} Kxg7 {+2.41/1 0.001s} 20. N@h5+ {+2.00/39 63s} Bxh5 {+3.19/24 24s} 21. Nxh5+ {+3.47/50 64s} Kf7 {+4.30/26 38s} 22. P@f2 {+3.24/52 64s} Q@g5 {+1.45/29 353s} 23. N@g7 {+2.82/68 64s} P@e5 {+1.22/22 51s} 24. B@c1 {+2.95/39 64s} Qxg7 {+3.18/28 53s} 25. Nxg7 {+3.09/40 65s} Kxg7 {+3.51/26 15s} 26. Q@h6+ {+3.44/52 65s} Kg8 {+4.11/28 31s} 27. Ng6 {+3.34/45 65s} N@d7 {+3.82/32 145s} 28. Nxe7+ {+2.76/61 66s} Qxe7 {+3.75/30 27s} 29. Bg5 {+2.58/62 66s} N@f6 {+4.60/26 40s} 30. B@g6 {+3.01/39 67s} hxg6 {0.00/32 91s} 31. Qxg6+ {+2.76/59 67s} B@g7 {0.00/32 24s} 32. P@h6 {+2.52/63 67s} Rf7 {0.00/33 38s} 33. c4 {+2.92/53 68s} d4 {0.00/29 228s} 34. Qxg7+ {+4.78/39 70s} Rxg7 {+2.82/1 0s} 35. hxg7 {+5.55/45 69s} Kxg7 {+2.82/23 42s} 36. B@h6+ {+5.64/54 70s} Kh8 {-0.67/26 40s} 37. Bxf6+ {+5.50/51 70s} Qxf6 {-2.75/28 80s} 38. Bg5 {+5.63/50 71s} Qg7 {-1.99/26 35s} 39. R@e7 {+5.72/49 72s} P@f7 {-3.66/29 161s} 40. N@h6 {+5.82/47 73s} N@d6 {+0.75/26 61s} 41. Rxd7 {+5.71/40 47s} Q@e8 {-3.52/27 67s} 42. Rxd6 {+7.06/36 48s} cxd6 {-5.20/25 36s} 43. N@f6 {+8.14/35 46s} Qef8 {-6.48/24 30s} 44. N@d7 {+9.63/40 45s} Qd8 {-4.26/26 13s} 45. Nxb6 {+9.92/43 44s} axb6 {-8.36/28 48s} 46. B@h4 {+10.26/29 43s} B@e7 {-8.62/25 30s} 47. Nh5 {+11.57/29 43s} Bxg5 {-16.58/25 30s} 48. Nxg7 {+11.80/29 42s} B@g6 {-17.85/26 30s} 49. Nxe6 {+12.90/37 42s} fxe6 {-17.90/24 14s} 50. Q@f7 {+14.02/29 41s} R@g7 {-21.50/26 39s} 51. Qxg6 {+14.73/24 40s} Rxg6 {-25.64/21 36s} 52. Nf7+ {+15.14/21 40s} Kh7 {-26.56/21 30s} 53. Nxd8 {+15.01/13 40s} Rxd8 {-30.14/18 30s} 54. Bxg5 {+16.85/15 39s} Rxg5 {-24.47/19 10s} 55. B@f6 {+17.60/18 38s} Q@g6 {-33.20/16 50s} 56. Bxd8 {+18.55/18 38s} N@h6 {-32.17/18 26s} 57. B@f6 {+20.36/19 38s} B@g7 {-37.25/16 34s} 58. Bxg5 {+22.83/22 37s} N@h5 {-38.50/18 30s} 59. Bxh6 {+23.79/12 37s} Bxh6 {-44.75/16 30s} 60. N@e7 {+24.61/12 36s} B@f7 {-M16/31 30s} 61. Nxg6 {+25.65/15 36s} Bxg6 {-M14/34 11s} 62. Q@e7+ {+24.38/11 36s} N@f7 {-M12/38 15s} 63. Q@e8 {+27.64/13 36s} N@h8 {-M12/38 21s} 64. R@g8 {+29.68/11 35s} N@f8 {-M10/49 16s} 65. Rxf8 {+31.01/9 35s} Bxf8 {-M10/54 15s} 66. Q7xf8 {+37.97/7 35s} Nxg3+ {-M10/61 15s} 67. fxg3 {+42.21/5 34s} R@g1+ {-M8/71 18s} 68. Rxg1 {+43.94/3 34s} P@g7 {-M6/97 19s} 69. Qg8+ {+34.15/7 34s} Kh6 {-M6/1 0.001s} 70. R@h4+ {+42.23/5 34s} Bh5 {-M4/1 0.001s} 71. R@h7+ {+81.41/3 34s} Kg6 {-M2/1 0.002s} 72. Qxg7# {+M1/1 50s, White mates} 1-0

Figure S16: Game 9 / 10

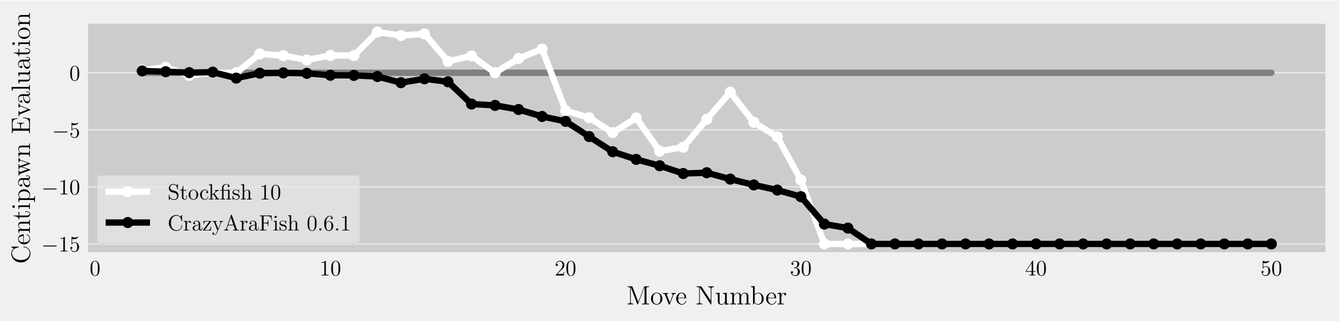

(a) Evaluation progression for both engines

[Event "CrazyAraFish-0.6.0-vs-SF10"]
[Site "Darmstadt, GER"]
[Date "2019.07.27"]
[Round "5"]
[White "stockfish-x86_64-modern 2018-11-29"]
[Black "CrazyAraFish-0.6.0"]
[Result "0-1"]
[PlyCount "102"]
[TimeControl "1800+30"]
[Variant "crazyhouse"]

1. g3 {book} d5 {book} 2. d4 {book} Nf6 {book} 3. Bg2 {book} Bf5 {-0.16/37 58s} 4. Nc3 {+0.23/30 174s} e6 {-0.09/26 59s} 5. Nf3 {+0.48/27 33s} Nc6 {-0.01/26 59s} 6. O-O {-0.23/28 120s} Be7 {-0.05/28 59s} 7. Ne5 {0.00/31 79s} O-O {+0.47/32 60s} 8. h3 {0.00/33 409s} h6 {+0.04/36 60s} 9. Nxc6 {+1.65/27 55s} bxc6 {+0.01/43 60s} 10. N@e5 {+1.50/26 41s} Qe8 {+0.05/42 60s} 11. Bf4 {+1.12/31 129s} Kh8 {+0.22/48 60s} 12. Qd2 {+1.52/25 25s} Kh7 {+0.23/50 61s} 13. Qc1 {+1.51/24 111s} N@g6 {+0.33/38 61s} 14. Nxg6 {+3.57/26 21s} fxg6 {+0.87/35 61s} 15. Bxc7 {+3.26/27 69s} N@g5 {+0.53/50 61s} 16. N@f4 {+3.41/26 101s} c5 {+0.79/41 61s} 17. Be5 {+1.00/27 337s} cxd4 {+2.74/40 62s} 18. Bxd4 {+1.48/25 11s} P@g4 {+2.86/50 62s} 19. hxg4 {0.00/25 137s} Nxg4 {+3.22/45 62s} 20. P@f3 {+1.25/26 31s} e5 {+3.83/43 62s} 21. fxg4 {+2.08/21 10s} Bxg4 {+4.26/48 63s} 22. Bxe5 {-3.32/24 258s} P@f3 {+5.59/30 63s} 23. exf3 {-3.94/23 128s} Bxf3 {+6.92/19 63s} 24. P@f7 {-5.25/24 97s} Qxf7 {+7.59/35 64s} 25. P@h5 {-3.96/20 17s} Bxh5 {+8.15/38 64s} 26. f3 {-6.87/23 62s} P@h3 {+8.83/29 65s} 27. Nxh3 {-6.52/20 12s} Nxh3+ {+8.76/31 65s} 28. Bxh3 {-4.08/20 18s} N@g5 {+9.32/33 65s} 29. P@g2 {-1.70/19 10s} Nxh3+ {+9.83/35 66s} 30. gxh3 {-4.34/21 37s} P@g2 {+10.28/31 66s} 31. Rf2 {-5.62/22 68s} B@d6 {+10.86/31 67s} 32. N@f6+ {-9.40/18 39s} Bxf6 {+13.26/15 68s} 33. Bxd6 {-15.35/17 30s} Bxc3 {+13.61/21 67s} 34. Bf4 {-17.50/17 30s} P@d2 {+16.76/21 68s} 35. Qd1 {-22.15/18 30s} Bxb2 {+17.33/21 69s} 36. Qxd2 {-24.37/16 30s} N@c4 {+19.66/18 69s} 37. Qe2 {-24.40/18 30s} N@d4 {+20.09/19 70s} 38. Qd3 {-31.53/17 30s} Bxa1 {+19.74/20 70s} 39. Rxg2 {-34.87/17 30s} Nxf3+ {+21.68/21 71s} 40. Kh1 {-53.50/18 27s} R@e1+ {+23.10/15 72s} 41. B@f1 {-44.17/18 26s} P@e4 {+24.32/16 47s} 42. N@h2 {-47.50/18 37s} exd3 {+26.75/17 46s} 43. P@e7 {-M18/21 30s} Nxh2 {+29.91/23 45s} 44. exf8=N+ {-M16/26 18s} Rxf8 {+30.27/13 45s} 45. R@h8+ {-M14/29 22s} Kxh8 {+31.60/9 44s} 46. P@e7 {-M12/37 21s} Rxf1+ {+29.56/11 43s} 47. N@g1 {-M10/53 13s} Rxg1+ {+29.11/9 42s} 48. Rxg1 {-M8/57 18s} N@f2+ {+31.25/7 42s} 49. Kg2 {-M6/67 18s} Bf3+ {+49.58/3 41s} 50. Kxh2 {-M4/75 26s} N@g4+ {+83.63/3 41s} 51. hxg4 {-M2/1 0.001s} R@h3# {+M1/1 60s, Black mates} 0-1

Figure S17: Game 10 / 10

REFERENCES

Silver, D., Huang, A., Maddison, C. J., Guez, A., Sifre, L., Van Den Driessche, G., et al. (2016). Mastering the game of Go with deep neural networks and tree search. *Nature* 529, 484–489

Silver, D., Hubert, T., Schrittwieser, J., Antonoglou, I., Lai, M., Guez, A., et al. (2017). Mastering chess and shogi by self-play with a general reinforcement learning algorithm. *arXiv preprint arXiv:1712.01815*

Smith, L. N. and Topin, N. (2019). Super-convergence: Very fast training of neural networks using large learning rates. In *Artificial Intelligence and Machine Learning for Multi-Domain Operations Applications* (International Society for Optics and Photonics), vol. 11006, 1100612
